# Intravascular lithotripsy versus rotational atherectomy for coronary atherosclerosis calcification: a systematic review and meta-analysis

**DOI:** 10.1186/s43044-026-00747-2

**Published:** 2026-05-21

**Authors:** Pirel Aulia Baravia, Faqrizal Ria Qhabibi, Isman Firdaus

**Affiliations:** 1 University of Brawijaya, Malang, Indonesia; 2 Pembuluh Darah Harapan Kita, Jakarta, Indonesia

**Keywords:** Intravascular lithotripsy, Rotational atherectomy, Coronary artery calcification, Percutaneous coronary intervention

## Abstract

**Background:**

Intravascular lithotripsy (IVL) is a novel calcium modification strategy with little evidence of efficacy compared to widely used rotational atherectomy (RA).

**Aims:**

We sought to compare the efficacy and safety level between IVL and RA in moderate-to-severe coronary artery calcification (CAC).

**Methods:**

We searched three databases until March 2025 for eligible observational studies and randomized controlled trials. The primary endpoint was major adverse cardiac event (MACE) while secondary endpoints were clinical outcomes, periprocedural complication, and procedural outcomes. Mantel-Haenszel risk ratio (RR) was used for dichotomous endpoints, and inverse variance mean difference (MD) for continuous variables, both with 95% confidence interval (CI).

**Results:**

Seven observational studies and two randomized clinical trials (RCT) with 968 patients were included; 418 IVL-treated patients and 550 RA-treated patients. IVL is associated with lower risk of MACE (IVL 1.5% vs. RA 5.3%; RR: 0.34 CI: 0.13–0.87) and all-cause mortality (IVL 1.6% vs. RA 5.4%; RR: 0.35 CI: 0.15–0.81) in in-hospital outcome. However, assessed with longer follow-up, IVL developed comparable risk of MACE (IVL 4.3% vs. RA 7.7%; RR 0.70 CI: 0.42–1.18) compared to RA while all-cause mortality remained lower in IVL (2.5% vs. 7.0%; RR: 0.41 CI: 0.22–0.78). No significant differences were observed in the risk of myocardial infarction (*p* = 0.58), nor in the risk of dissection (*p* = 0.52), perforation (*p* = 0.22), or slow/no-reflow (*p* = 0.07). IVL likewise demonstrated comparable procedural performance, including procedural success, angiographic success, fluoroscopy duration, and procedural time.

**Conclusion:**

IVL has similar outcomes to RA in terms of procedure outcomes and periprocedural complications. However, IVL is associated with lower all-cause mortality and MACE specifically in short term outcomes.

**Supplementary Information:**

The online version contains supplementary material available at 10.1186/s43044-026-00747-2.

## Introduction

One-third patients who underwent percutaneous coronary intervention (PCI) have been revealed to have coronary artery calcification (CAC) [1–3]. CAC is an advanced phase of atherosclerosis that increases the stiffness of the artery resulting in reduced vasomotor response and decreased myocardial perfusion [4]. Due to its low compliance, CAC negatively affects coronary intervention by impending device crossing and stent expansion resulting in higher risk of having stent thrombosis and restenosis. [2] This mechanism will increase other complications such as major adverse cardiac event (MACE) [5, 6] and periprocedural complication like dissection, perforation, and slow/no reflow. [7, 8]. Due to this feature, CAC is still considered a challenging case for interventional cardiologist [9, 10].

Currently, there are multiple strategies to modify CAC including balloon-based techniques such as high-pressure balloon and athero-ablative technology like rotational atherectomy (RA) for example [11]. However, high pressure balloon is considered insufficient to fracture calcium and achieve vessel expansion and, furthermore, can lead to barotrauma-related dissection or perforation [2]. Moderate-to-severe coronary calcification is generally managed with athero-ablative techniques, particularly RA, rather than high-pressure balloon strategies and evidence from meta-analysis indicates that RA achieved superior stent delivery and angiographic expansion [12]. This result justifies the widely used of RA for decades to treat more severe coronary artery calcification [8–10]. However, at the risk of developing procedural complications, both techniques are still showing similar results [2, 13].

IVL is a novel technique that has a similar concept with kidney stone treatment, adapted for the management of CAC [14, 15]. This principle is used to crack the plaque without resulting damage to the vascular wall or adjacent soft tissue. Due to its groundbreaking principle, there are several studies that have been conducted and stated that IVL has shown high success rate with excellent early post-PCI outcomes and long-term clinical outcomes [16, 17]. With this being a hot topic in cardiac intervention circles, remembering the challenge of CAC, we conducted this meta-analysis to comprehensively compare IVL and RA in moderate-to-severe CAC, including recent studies, assessing the safety level in terms of periprocedural complications that comprised of perforation, dissection, and slow/no reflow, clinical outcomes consisted from MACE, myocardial infarction, and all-cause mortality, and efficacy level from procedural outcomes that cover procedural success, angiographic success, fluoroscopy time, and procedural time.

## Method

### Data sources and search strategy

A comprehensive literature search was conducted independently by two authors on PubMed, Embase, and Cochrane Library to identify relevant publications. The last electronic search was performed on March, 2025. We also reviewed the reference list of the original trials, prior meta-analysis, and review articles. There were no restrictions on language. For the search strategy, we used, in various relevant combination, MeSH terms and keywords pertinent to the related topic: (“intravascular lithotripsy” OR “IVL” OR “shockwave lithotripsy” OR “coronary lithotripsy”) AND (“Rotational atherectomy” OR “RA” OR “rotablator” OR “rotational ablation”). The establishment of inclusion and exclusion criteria was performed before the literature search, adhering to the PICOS approach (Supplemental Table 1). Subsequently, the obtained results are purged of duplicates and evaluated according to the eligibility criteria. The registered study protocol is available on PROSPERO (CRD42024619925). The meta-analysis findings were reported according to the Preferred Reporting Items for Systematic Reviews and Meta-Analyses (PRISMA). No additional ethical clearance is required since this study is based on a secondary literature analysis of published observational studies.

### Selection criteria and data extraction

We included observational studies and RCTs that compare the clinical outcomes, periprocedural complication, or procedural characteristics of IVL vs. RA for moderate-to-severe CAC. Moderate calcification is defined as radiopaque densities not only with cardiac motion before a contrast injection, while severe calcification is defined as radiopaque densities without cardiac motion before contrast injection. We excluded ineligible study designs, review articles, case reports, case series, and in-vitro studies. Duplicate reports, post-hoc analyses, and irretrievable data after contacting the leading author also being excluded. Two investigators individually retrieved data from the selected observational studies and RCTs utilizing a standardized computerized form. Disputes between the two investigators were settled by consensus with the involvement of a third investigator. Extracted data from selected studies including first author’s name, year of publication, follow-up duration, study center, study design, lesion target, device types, imaging technique, and sample size. The corresponding authors of the included studies were contacted if any data was missing.

### Assessment of risk of bias and quality of evidence

The quality assessment of each included study was performed independently by two investigators under the supervision of the senior author. This study employed the Newcastle-Ottawa Scale (NOS) to assess bias risk and produce definitive estimates of the effects on outcomes [18]. High-quality studies concerning NOS were identified by achieving a rating of 3 or 4 stars in the selection domain, 1 or 2 stars in the comparability domain, and 2 or 3 stars in the outcome/exposure domain [19] (Supplemental Table 2). The quality study of included RCT was assessed using the Cochrane risk assessment tool of bias [20] (Supplemental Table 3). Studies were classified into low risk, unclear risk, or high risk of bias.

### Outcomes measures

The primary endpoint of this study was major adverse cardiac event (MACE). Secondary endpoints of this study are safety and efficacy level. Safety level was assessed from clinical events such as myocardial infarction and all-cause mortality, together with periprocedural complication comprising dissection, perforation, and slow/no-reflow. Efficacy level was assessed from procedural success, angiographic success, fluoroscopy time, and procedural time. The definitions of outcomes in the included studies are provided in Supplemental Table 4.

### Statistical analysis

A meta-analysis was conducted across all variables related to outcome. The data were examined utilizing random effects analysis in Review Manager (RevMan) software (Version 5.4. Copenhagen: The Nordic Cochrane Centre, The Cochrane Collaboration). Summary estimates for continuous variables were reported as mean difference (MD), and for categorical variables were reported as risk ratios (RRs) with an exception of angiographic success and procedural success, they were reported as odd ratios (ORs). These data were incorporated into the Mantel-Haenszel method with 95% confidence intervals (CI). The Q test was utilized to evaluate heterogeneity, with results shown as I^2^ values. Statistical heterogeneity across the included studies was ascertained using I^2^ statistics, such that I^2^ statistic values < 25%, 25% to 50%, and > 50% corresponded to a low, moderate, and high degree of heterogeneity, respectively [20]. A 2-tailed *P* value of < 0.05 was set for statistical significance. Publication bias was not assessed because of the few numbers of the included studies [21]. Subgroup analysis was done according to study design (RCT vs. observational study).

## Result

### Study selection, characteristics, and risk of bias

**Fig. 1:**
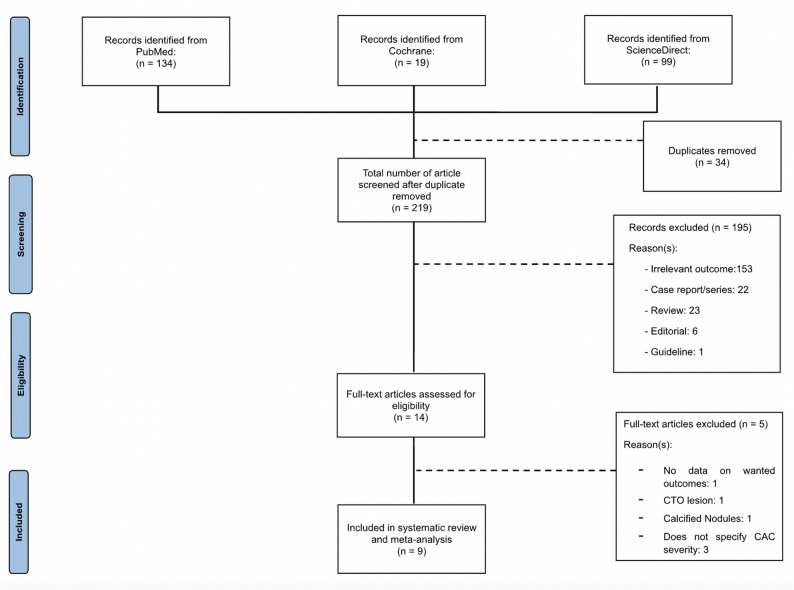
Study flow chart. A description of the study selection process for the systematic review and meta-analysis.* CAC* coronary artery calcification,* CTO* chronic total obstruction

The study selection flow is provided in Fig. 1. The final analysis included seven observational studies [7–9, 11], [22–24] and two randomized controlled trials [10, 25] with 968 patients; 418 patients were in the IVL arm and 550 patients were in the RA arm. One study was excluded due to no wanted data provided [26], one study was excluded because the study was done in chronic total occlusion [27], one study was done specifically in calcified nodules [28], and another two studies were also excluded because there was no definition on CAC severity [29, 30]. The median follow-up period of included studies is 3.5 months. The characteristics of the included studies and patients appear in Tables 1 and 2. All included studies used The Shockwave Medical as the IVL device. Meanwhile, for RA, 7 studies used Rotablator from Boston Scientific and 2 studies did not specify. There characteristics of the included studies and patients appear in (Table 1, Supplemental Tables 5, and Supplemental Table 4). The quality of the included studies is provided in (Supplemental Table 6). According to the NOS, seven studies were assessed as low risk of bias (Supplemental Table 2). Two RCTs were assessed as low risk based on the RoB tool (Supplemental Table 3).

### Safety outcomes

**Fig. 2:**
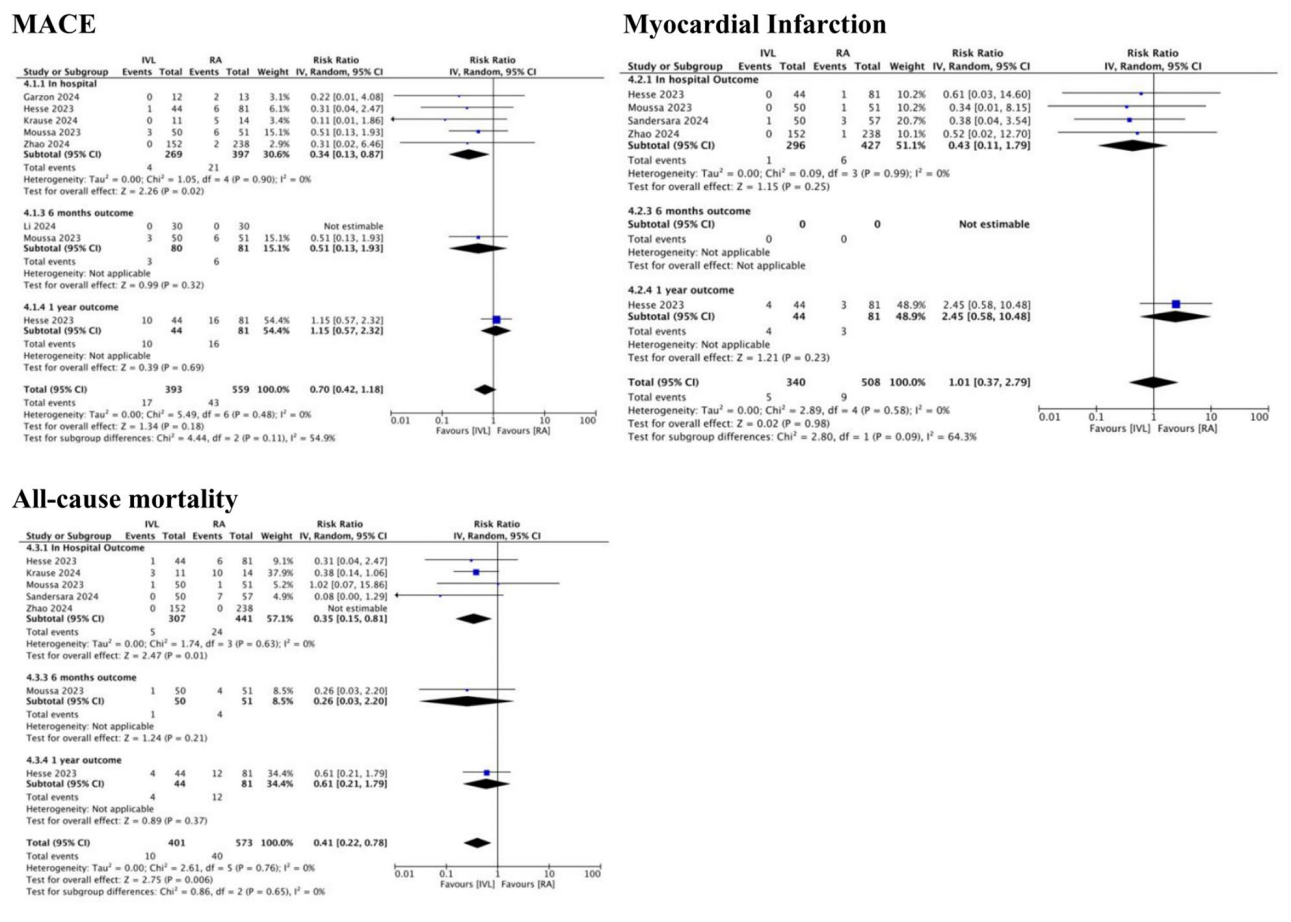
Forest plot for primary outcomes. A forest plot for the risk of major adverse cardiac event, myocardial infarction, and all-cause mortality evaluated from in-hospital, 6 months, and 1-year follow up.* MACE* major adverse cardiac outcome,* M-H* Mantel-Haenzel

Based on in-hospital outcome, IVL has significantly lower risk of developing MACE (IVL 1.5% vs. RA 5.3%; RR: 0.34 CI: 0.13–0.87; I^2^ = 0%) and all-cause mortality (IVL 1.6% vs. RA 5.4%; RR: 0.35 CI: 0.15–0.81; I^2^ = 0%) compared to RA. However, MACE was found to be comparable between IVL and RA in longer follow-up (IVL 4.3% vs. RA 7.7%; RR 0.70 CI: 0.42–1.18; I^2^ = 0%) with the risk of all-cause mortality remain lower in IVL (IVL 2.5% vs. RA 7.0%; RR: 0.41 CI: 0.22–0.78; I^2^ = 0%)(Central Illustration) (Fig. 2).

### Periprocedural complications and procedural outcomes

**Fig. 3:**
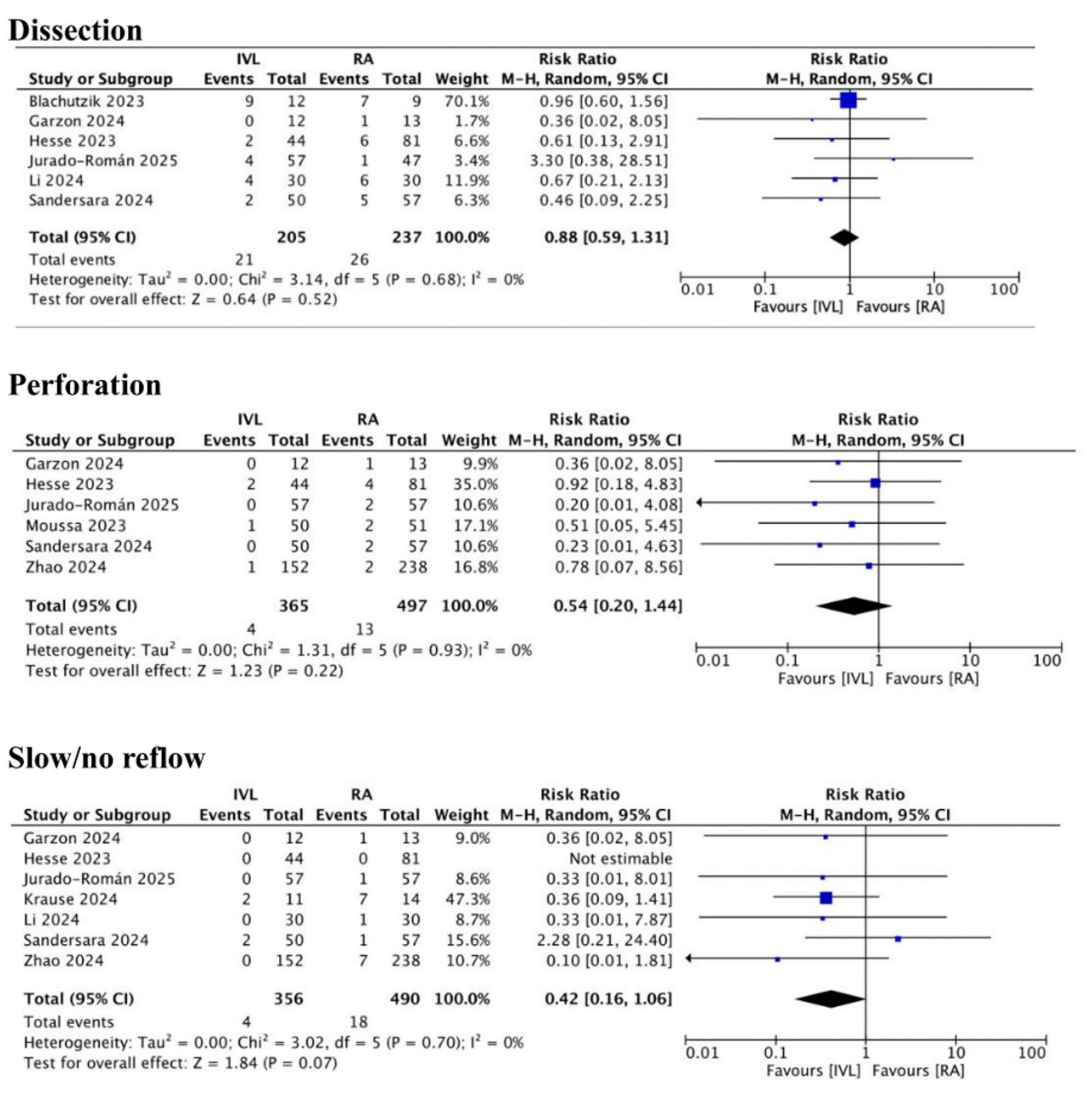
Forest plot for periprocedural complication. A forest plot for the risk of dissection, perforation, and slow/no reflow. Abbreviation as in Fig. 2

**Fig. 4:**
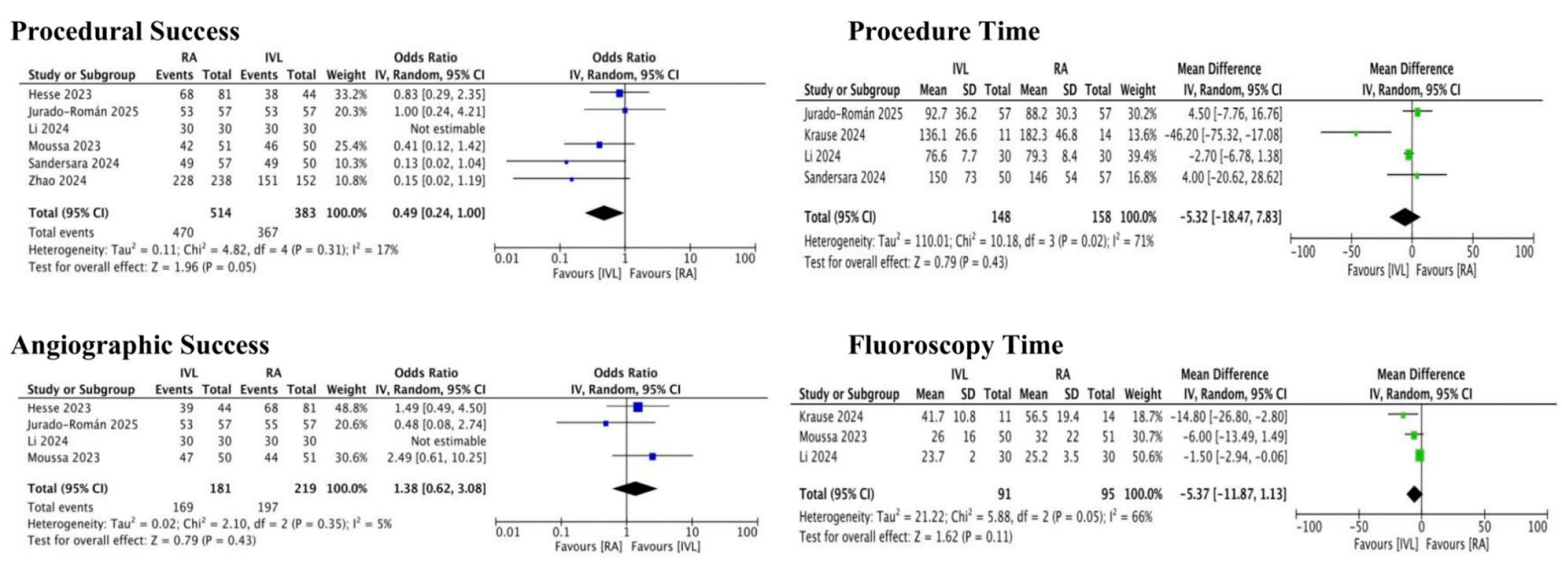
Forest plot for procedural characteristic. A forest plot of procedural success, angiographic success, procedure time, and fluoroscopy time.* IV* inverse variance

There were no differences in all periprocedural complications from the primary analysis (Central Illustration, Fig. 3). This result remain unchanged with subgroup analysis between observational studies and RCTs(Supplemental Fig. 1). On procedural outcomes, there was no difference in both procedural success (IVL 91.4% vs. RA 95,8%; OR: 0.49 CI: 0.24–1.00; I^2^ = 17%) and angiographic success (IVL 93.4% vs. RA 89.4%; OR: 1.38 CI: 0.62–3.08; I^2^ = 5%). There were no differences between IVL and RA regarding procedural time (MD −5.32; CI: −18.47 to 7.83; *P* = 0.43; I^2^ = 71%) and fluoroscopy time (MD −5.37; CI: −11.87 to 1.13; *P* = 0.11; I^2^ = 66%) (Central Illustration, Fig. 4). Subgroup analysis between observational and RCT found comparable outcomes in procedural outcomes except procedural success. Procedural success was significantly higher favoring IVL compared to RA in observational studies (IVL 96.3% vs. RA 91.3%; OR 0.40 CI: 0.17–0.93; I^2^ = 24%) but comparable result between IVL and RA on RCT(Supplementary Fig. 2).

### Sensitivity analysis

**Fig. 5:**
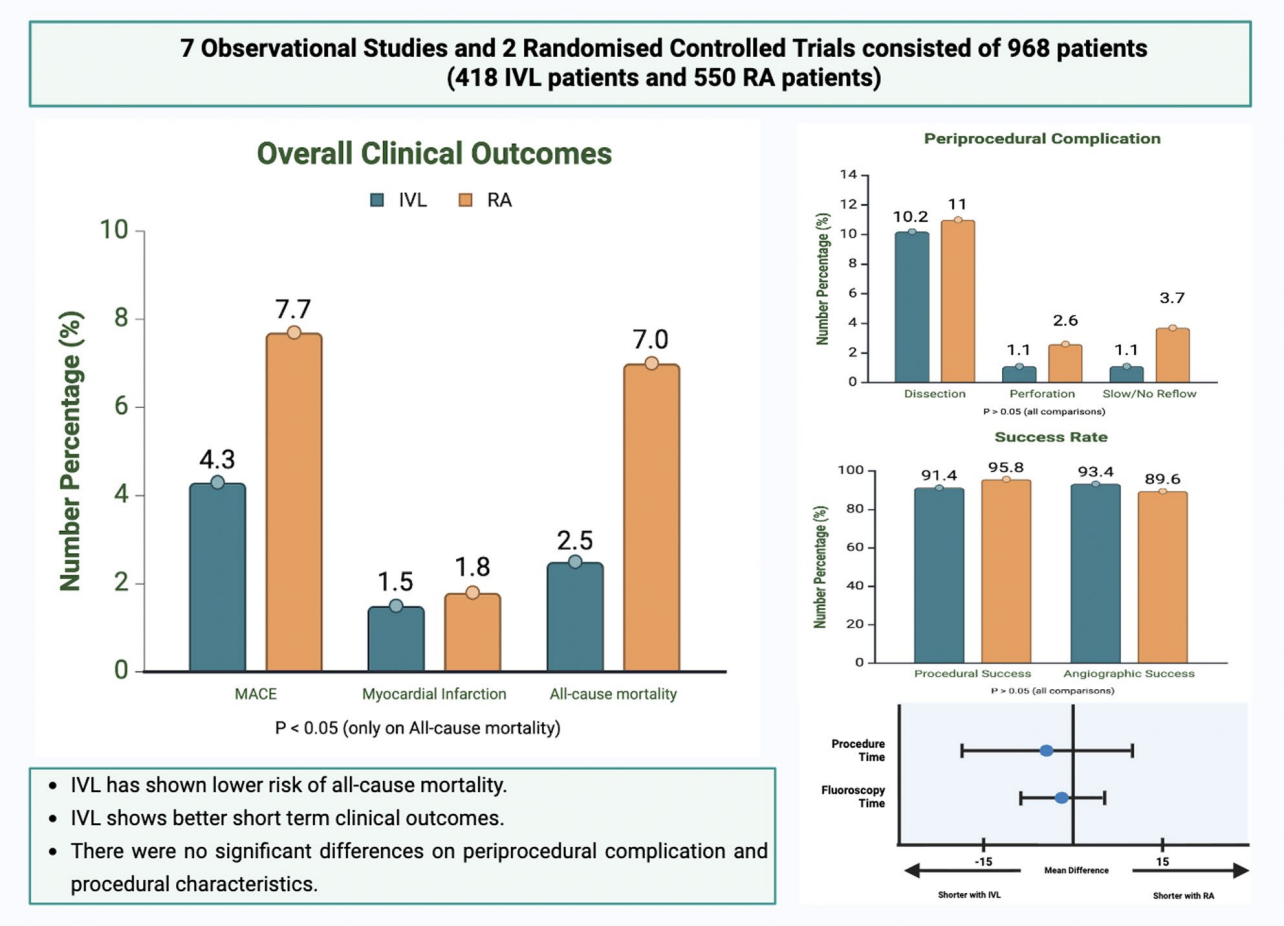
Central illustration. Summary overall clinical outcomes, periprocedural outcome, and procedural characteristics as secondary outcome.* IVL* intravascular lithotripsy,* MACE* major adverse cardiac event,* RA* rotational atherectomy

In a sensitivity analysis after omitting a study with undefined calcification severity, MACE was influenced by Hesse et al., although the remaining studies also contributed to the overall estimate. For slow/no reflow, the exclusion of Krause et al. did not change the effect estimate, whereas omission of Hesse et al. did, suggesting their study influenced this endpoint. For all, remaining endpoints, the exclusion of either Hesse et al. or Krause et al. did not alter the overall effect estimates (Fig. 5).

The outcome of fluoroscopy time and procedure time was also assessed where in term of fluoroscopy time, the exclusion of Li et al. change the *p*-value between the original pooled and leave-one-out analysis suggesting that Li et al. materially influenced the overall effect estimate. In contrast for procedure time, although the heterogeneity also exceeded 60%, the leave-one-out analysis demonstrated that omitting any individual study did not change the statistical significance relative to the original pooled estimate.

We also conducted the sensitivity analysis based on study design, however due to limited number of study, the only analysis that can be done was dissection where there was no signle study that changed the significance of *p*-value from the original pooled outcome (Supplementary Fig. 6).

## Discussion

This meta-analysis comprehensively covers not only clinical outcome but also periprocedural complications and procedural outcomes on IVL vs. RA for moderate-to-severe CAC. From 7 observational studies and 2 RCTs, we found:

- IVL was associated with lower all-cause mortality with similar MACE and myocardial infarction at a mean follow-up of 4.2 months and associated with lower risk of developing MACE and all-cause mortality in in-hospital outcome.
- There were no significant differences on dissection, perforation, and slow/no reflow evaluated after procedure.

All included studies use the same IVL device by Shockwave Medical which is a single-use, disposable that contains an emitter throughout the shaft of the balloon. This emitter produces rapid vapor bubbles that initiate acoustic pressure through rapid formation that will be delivered circumferentially in an unfocused manner. This dispersed shockwave will result low energy density in the targeted area, resulting in energy that is enough to create superficial to deep calcium fracture but just below the threshold of causing soft tissue damage [2]. In addition to our findings, the pooled analysis of DISRUPT CAD I-IV studies provide important context regarding the performance of IVL in severely calcified coronary lesions. Across these studies, IVL demonstrated consistent procedural success and favorable safety outcomes. For instance, the 30-day MACE rates remained low across all studies: 5% in DISRUPT CAD I [31], 7.6% in CAD II [16], 7.8% in CAD III [32], and 6.3% in CAD IV [33]. Angiographic success was consistently high, ranging 96.4% to 100% accompanied by procedural success rates between 92.2% and 95%, with stent delivery achieved in 100% cases.

On the other hand, RA produces lumen enlargement with an elliptical burr that rotates at very high speed removing plaque and facilitating dilation. This makes the luminal surface become smooth with cylindrical geometry [34]. An RA needs a guidewire to help the burr tip coaxis with the vessel lumen and should be positioned distal to the target lesion avoiding small side branches and distal narrow vasculature. However, there are several problems with RA. First, despite using a guidewire, a highly tortuous or angulated segment can disrupt the axis of the burr creating a wire bias that may predispose to tissue injury. Second, the heat produced from the friction of the burr and the plaque will cause thermal injury. Together with the debris, this can contribute to periprocedural complications associated with excessive deceleration [34]. Regarding the existing problem, the Floppy wire from RotaWire provides more flexibility with the expectation to reduce wire bias and permits ablation with greater curvature of angulation. To minimize thermal injury, flush solutions like heparinized saline and RotaGlide lubricant have been shown to reduce heat generation and prevent sudden deceleration [35].

To treat severe CAC, IVL has several advantages compared with other calcium modification strategies. As mentioned before, IVL does not rely on high energy whereas other balloon-based therapies require high-pressure balloons that can potentially cause soft tissue damage due to barotrauma. IVL also has promising coverage due to its circumferential shockwave distribution with the ability to initiate superficial-to-deep calcium fractures. This is theoretically superior compared with athero-ablative therapy (rotational or orbital atherectomy) that has a limitation to modify deep calcium. Not forgetting the wire bias that athero-ablative has that can cause incomplete calcium modification which eventually negatively affect procedural safety [2].

To the best of our knowledge, there are two meta-analyses that cover the same topic. According to meta-analysis conducted by Gupta et al. [36], IVL and RA were not significantly different in terms of periprocedural complication comprising coronary perforation, target vessel revascularization, and stent thrombosis. This resulting consistent result in clinical outcomes where IVL and RA is comparable in MACE, myocardial infarction, and death. Fluoroscopy time is also shown comparable between IVL and RA. However, their meta-analysis only covered five studies. With recent publication, the result has changed where the risk of all-cause mortality is lower in IVL compared to RA and, to be precise, the present meta-analysis showed that MACE and all-cause mortality is lower in shorter time follow-up. Other meta-analysis by Suruagy-Motta et al. [37] showed that procedural success was higher in IVL group is consistent with our result especially in observational study.

The present meta-analysis showed that despite overall procedural success being comparable between IVL and RA group, subgroup analysis showed IVL has higher procedural success in the observational group compared to RCT group. This data affect the clinical outcomes of this meta-analysis due to; First the two RCT did not provide the clinical outcomes, hence all clinical outcomes consisted of observational studies. Second, despite high variability of procedural success in all included studies, the main definition is angiographic success without in-hospital MACE, this is the reason IVL group has lower risk of MACE compared to RA.

For all-cause mortality being higher in both short follow up and overall outcomes is due to Krause et al. [24] has higher weight on forest plot compared to other included studies. Krause et al. conducted the research in high risk patients in which percutaneous mechanical circulatory support is used where the patient is at risk of hemodynamic compromise due to impaired left ventricular ejection fraction. According to the report of their cohort studies, the patient in RA group is more unstable compared to the IVL group as a higher rate of vasopressor was needed to maintain stable hemodynamic. Patients in the RA group also found to have more complex lesions compared to IVL as Syntax score is higher together with longer calcified lesions. In this study, a bailout strategy was found higher in the RA group compared to IVL group which was associated with higher in-hospital death and the occurrence of slow reflow phenomenon.

Overall, the included studies demonstrated generally comparable baseline demographics and cardiovascular risk profiles between group. However, Mousa et al. [23] demonstrated a notable difference in hypertension prevalence, which was higher in the IVL group comapred with the RA group, suggesting a potentially higher baseline cardiovascular risk profile in the IVL cohort.

In addition to this topic, there is an ongoing RCT that compares peri-procedural myocardial infarction in IVL and RA for calcified coronary artery lesions. SONAR (ShOckwave ballooN or Atherectomy with Rotablation in calcified coronary artery lesion; NCT05208749) is an open-label, multicenter, investigator-initiated, prospective that will randomize 170 patients with moderate to severe calcified coronary lesions. All the patients who meet all inclusion criteria will be randomized 1:1 to IVL or RA via the REDCap randomization module where clinical outcomes will be evaluated in the 1 month and 1 year after procedure as secondary outcomes.

**Table 1:** Baseline characteristics of the study population

First author	Year	Mean age (year)		Male (%)		Hypertension (%)		Diabetes (%)		Hyperlipidemia (%)	
		IVL	RA	IVL	RA	IVL	RA	IVL	RA	IVL	RA
Garzon	2024	78 (71–81)	72 (65–83)	83.3	84.6	75.0	76.9	53.8	50.0	75.0	76.9
Sandersara	2024	71 ± 12	75 ± 10	68	59.6	92	93	58	59	100	100
Hesse	2023	74.2 ± 8.5	74.3 ± 8.4	79.5	81.5	72.7	76.5	40.9	32.1	59.1	48.1
Mousa	2023	73.7 ± 8.9	72.8 ± 8.7	68	59	**76**	**55**	40	27	67	60
Li	2024	65.0 ± 3.8	68.0 ± 5.9	46	56	96	90	66	76	83	86
Zhao	2024	65 ± 8	66 ± 8	75.0	65.5	64.5	68.9	41.4	47.9	-	-
Jurado-Román	2025	70.7 ± 8.2	70.8 ± 8.2	78.6	80.7	78.6	77.2	48.2	45.6	73.2	68.4
Krause	2024	74.4 ± 7.9	76.6 ± 8.4	32	44	40	56	20	24	36	40
Blachutzik	2023	76.3 ± 7.0	81.4 ± 6.9	83.3	77.8	100	100	33.3	33.3	83.3	66.7

Values are mean ± SD, %, or median (IQR). IVL = intravascular lithotripsy; RA = rotational atherectomy

**Table 2:** Characteristics of included studies

First author	Year	Follow up	Center	Study design	Lesion target	Device type		Guidance	Sample size		
						IVL	RA		IVL	RA	Total
Garzon	2024	30 days	Single Center (Brazil)	Retrospective Observational	Any Severe Calcification	Shockwave C2 balloon (Shockwave Medical Inc., Santa Clara, California, USA)	RA (Boston Scientific, Nantucket, Massachusetts, USA)	IVUS	12	13	25
Sandersara	2024	30 days	Single Center (United States of America)	Retrospective Observational	Severely calcified de novo or post CABG distal LM stenosis who underwent PCI and were treated with IVL or RA	IVL (Shockwave Medical)	RA (Boston Scientific)	IVUS	50	57	107
Hesse	2023	1 year	Single Center (United Kingdom)	Retrospective Observational	Unprotected LMCA (stenosis without a patent bypass graft to LAD and/or LCx and severe coronary artery calcification	Shock C2 balloon-based coronary catheter system (Shockwave Medical Inc., Santa Clara, California, USA)	does not specify	Intravascular Imaging either IVUS or OCT	44	81	125
Mousa	2023	6 months	Single Center (Netherland)	Retrospective Observational	Severe coronary artery calcification (CAC)	Shockwave Intravascular Lithotripsy Coronary System (Shockwave Medical, Santa Clara, California).	Rotablator system (Boston Scientific, Natick, Massachusetts)	does not specify	50	51	101
Li	2024	6 months	Single Center (China)	Retrospective Observational	Severe coronary artery calcification	Shockwave C2 disposable intravascular catheter (Shockwave Medical, Santa Clara, CA, USA)	Rotablator TM (model H802220200381; Boston Scientific Corporation, Shanghai, China)	IVUS	30	30	60
Zhao	2024	In hospital only	Single Center (China)	Retrospective Observational	Severe Coronary artery calcification (CAC)	The Shockwave Medical (Santa Clara, CA, USA)	Rotational atherectomy (Boston)	Either IVUS or OCT	152	238	390
Jurado-Román	2025	12 months	Multicenter	RCT	Moderate to severe artery calcification	Shockwave balloon (Shockwave Medical)	RotaPro System (Boston Scientific)	OCT	57	57	114
Krause	2024	N/A	Single Center (Germany)	Retrospective Observational	Severe coronary artery disease due to complex coronary anatomy, calcified stenoses and corresponding previous illnesses who are not suitable for surgical care	IVL (Shockwave^®^ Medical Inc. Corporate Headquarters, 5403 Betsy Ross Drive, Santa Clara, CA 95054, USA)	RA (Boston Scientific^®^ World Headquarters, 300 Boston Scientific Way Marlborough, MA, USA)	does not specify	11	14	25
Blachutzik	2023	In hospital only	Multicenter	RCT	Clinically significant and severely calcified coronary lesions	IVL (Shockwave^®^ Medical Inc. Corporate Headquarters, 5403 Betsy Ross Drive, Santa Clara, CA 95054, USA)	Does not specify	OCT	12	9	21

### Limitation

The current systematic review and meta-analysis has several limitations that should be acknowledged. First, there are only two studies that cover 1-year follow up with the mean of 4.2 months on clinical outcome. Second, the diversity of patient conditions might affect the heterogeneity of this study that can cause clinical heterogeneity. Third, the total number of patients included in this meta-analysis is relatively low, which can make the study that has a larger portion of population might skew the result. Fourth, confounding factors cannot be excluded in the observational study, especially given inherent bias associated with observational study. Despite these limitations, this study represents the first meta-analysis comparing IVL and RA for moderate-to-severe coronary artery calcification and offers valuable data and insight on the outcome of IVL as a novel calcium modification technique.

## Conclusion

Based on the results of this meta-analysis, IVL compared to RA was associated with lower risk of all-cause mortality and MACE specifically at short follow up explained with higher procedural success. However, given the very low number of mortality events and predominance of observational study, this finding should be interpreted as hypothesis-generating rather than definitive. Larger randomized controlled trials with longer follow-up comparing IVL and RA are needed to provide more conclusive evidence.

### Clinical perspective

#### What is known

RA has been extensively used for modifying severe calcified lesions due to its effectiveness in stent delivery and angiographic expansion. However, the device has inherent limitations that can lead to significant procedural complications. In contrast, IVL introduces a novel approach, theoretically addressing the challenges associated with RA. However, current evidence supporting its superiority remains insufficient.

#### What is new

This meta-analysis, which included 7 observational studies and 2 RCTs involving 968 patients, found no significant differences in periprocedural outcomes and majority of procedural outcomes. However, IVL was associated with better clinical outcomes, particularly lower all-cause mortality and in-hospital MACE rates.

#### What is next

Future research involving a larger patient population and extended follow-ups is necessary to determine the optimal device for treating moderate-to-severe CAC.

## Supplementary Information

Below is the link to the electronic supplementary material.

Supplementary Material 1

## Data Availability

No datasets were generated or analysed during the current study.

## Abbreviations

CI: Confidence interval
CAC: Coronary artery calcification
IVL: Intravascular lithotripsy
MACE: Major adverse cardiac event
MD: Mean difference
NOS: Newcastle-Ottawa scale
OR: Odds ratio
PCI: Percutaneous coronary intervention
PRISMA: Preferred reporting items for systematic reviews and meta-analyses
RCT: Randomized clinical trial
RR: Risk ratio
RA: Rotational atherectomy
