# Supplementary material for: Intravascular lithotripsy versus rotational atherectomy for coronary atherosclerosis calcification: a systematic review and meta-analysis
Source: Egypt Heart J. 2026 May 21;78:36. doi: 10.1186/s43044-026-00747-2 (PMC13194918; doi:10.1186/s43044-026-00747-2)
Supplement: Supplementary file 1 — Supplementary Material 1 [file 43044_2026_747_MOESM1_ESM.docx]

**Supplemental Appendix Table of Contents**

| **Item** | **Page Number** |
| --- | --- |
| **Supplementary Figure 1. Subgroup Forest Plot for Periprocedural Complication** |  |
| **Supplementary Figure 2. Subgroup Forest Plot for Procedural Outcomes** |  |
| **Supplementary Figure 3. Sensitivity Analysis of Safety Outcomes** |  |
| **Supplementary Figure 4. Sensitivity Analysis of Periprocedural Complication** |  |
| **Supplementary Figure 5. Sensitivity Analysis of Procedural Outcomes** |  |
| **Supplementary Figure 6. Sensitivity Analysis of Dissection based on Study Design** |  |
| **Supplementary Table 1. PICOS** |  |
| **Supplementary Table 2. Newcastle-Ottawa Scale** |  |
| **Supplementary Table 3. RoB 2 Tool** |  |
| **Supplementary Table 4. Inclusion and exclusion criteria and definition of outcomes** |  |
| **Supplementary Table 5. Characteristics of Included Studies** |  |
| **Supplementary Table 6. GRADE Assessment** |  |

**Supplementary Figures**

**Supplementary Figure 1. Subgroup Forest Plot for Periprocedural Complication**


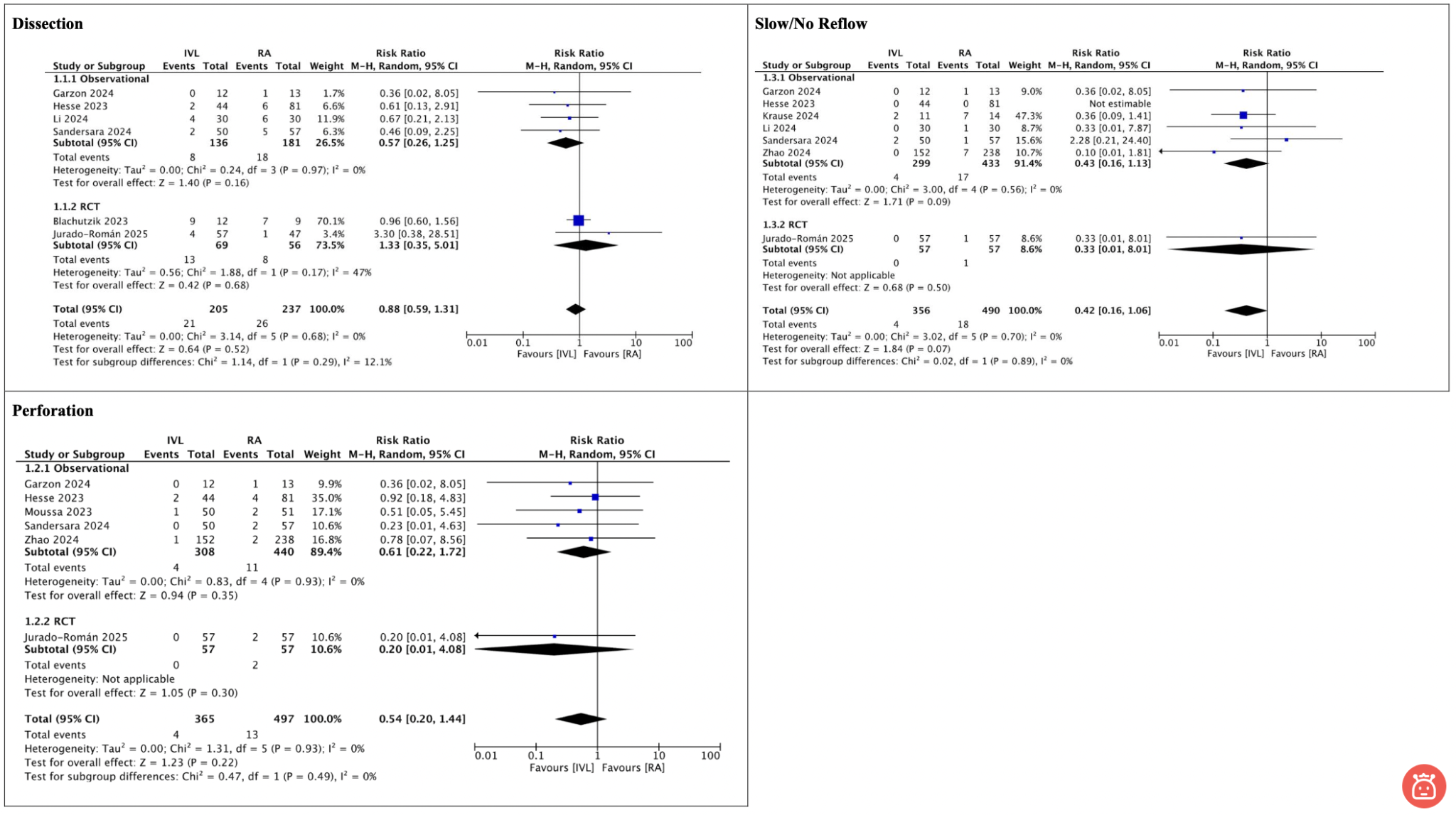


**Supplementary Figure 2. Subgroup Forest Plot for Procedural Outcomes**

**
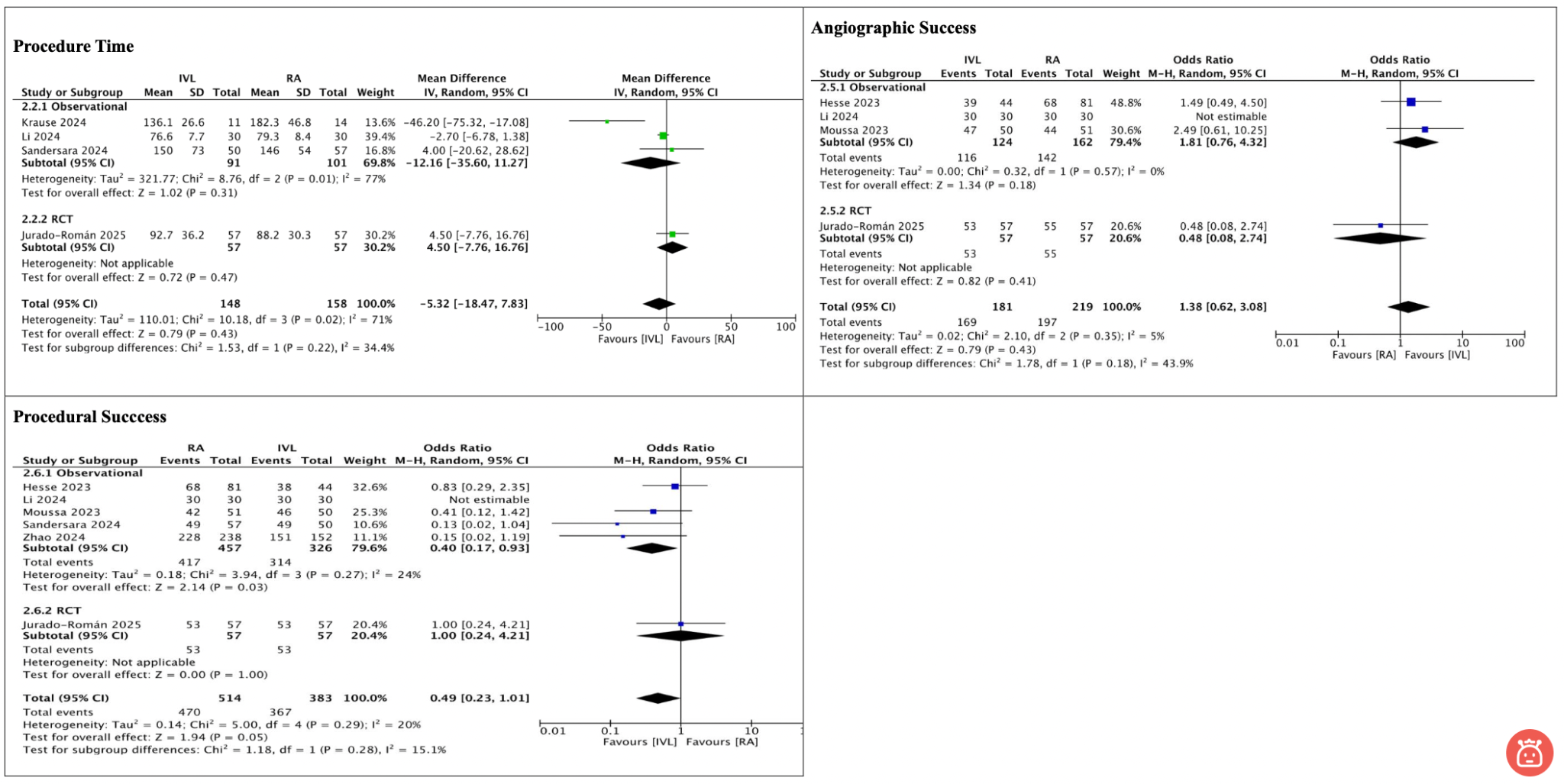
**

**Supplemental Figure 3. Sensitivity Analysis of Safety Outcomes**

**
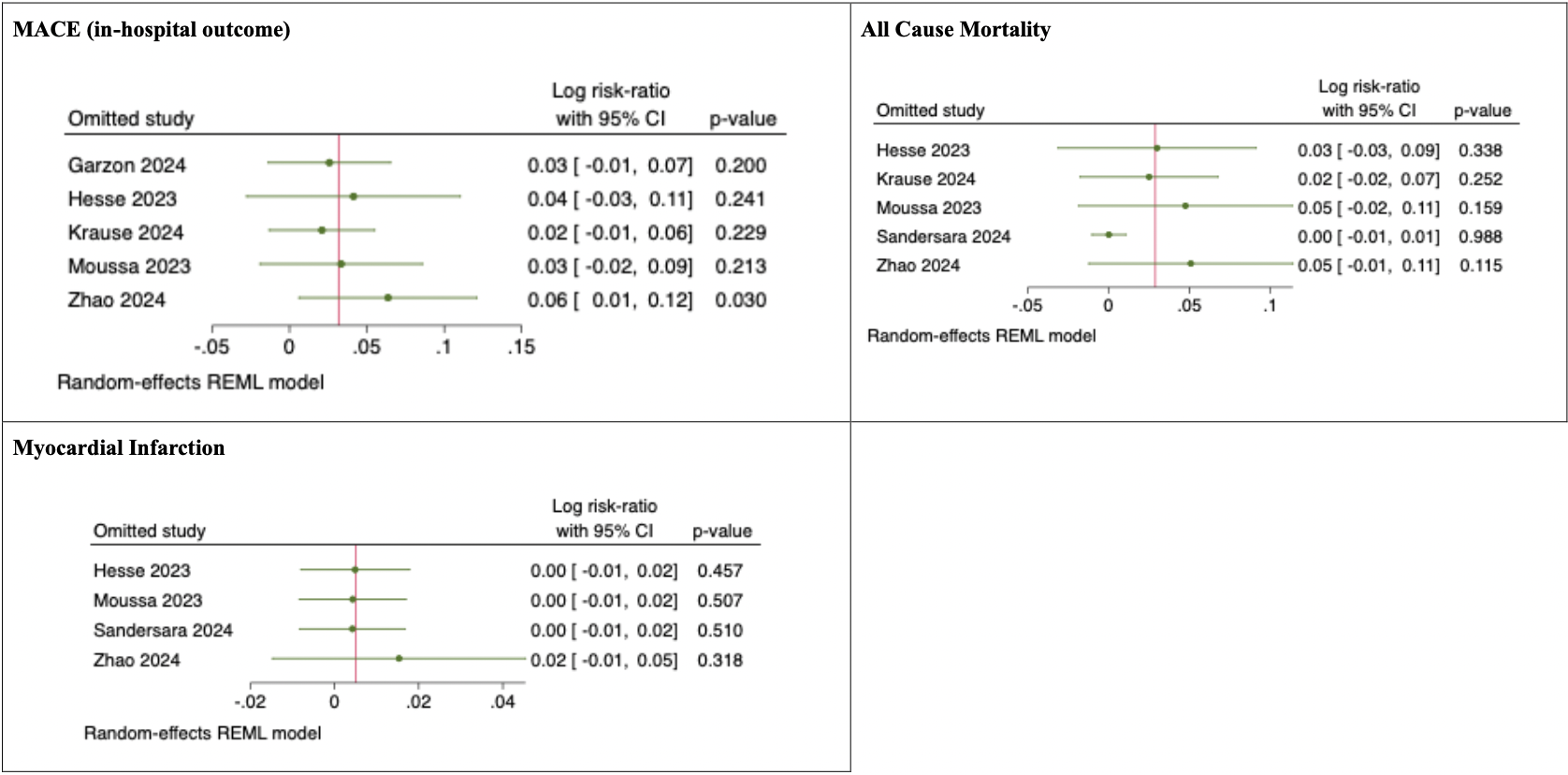
**

**Supplementary Figure 4. Sensitivity Analysis of Periprocedural Complication**

**
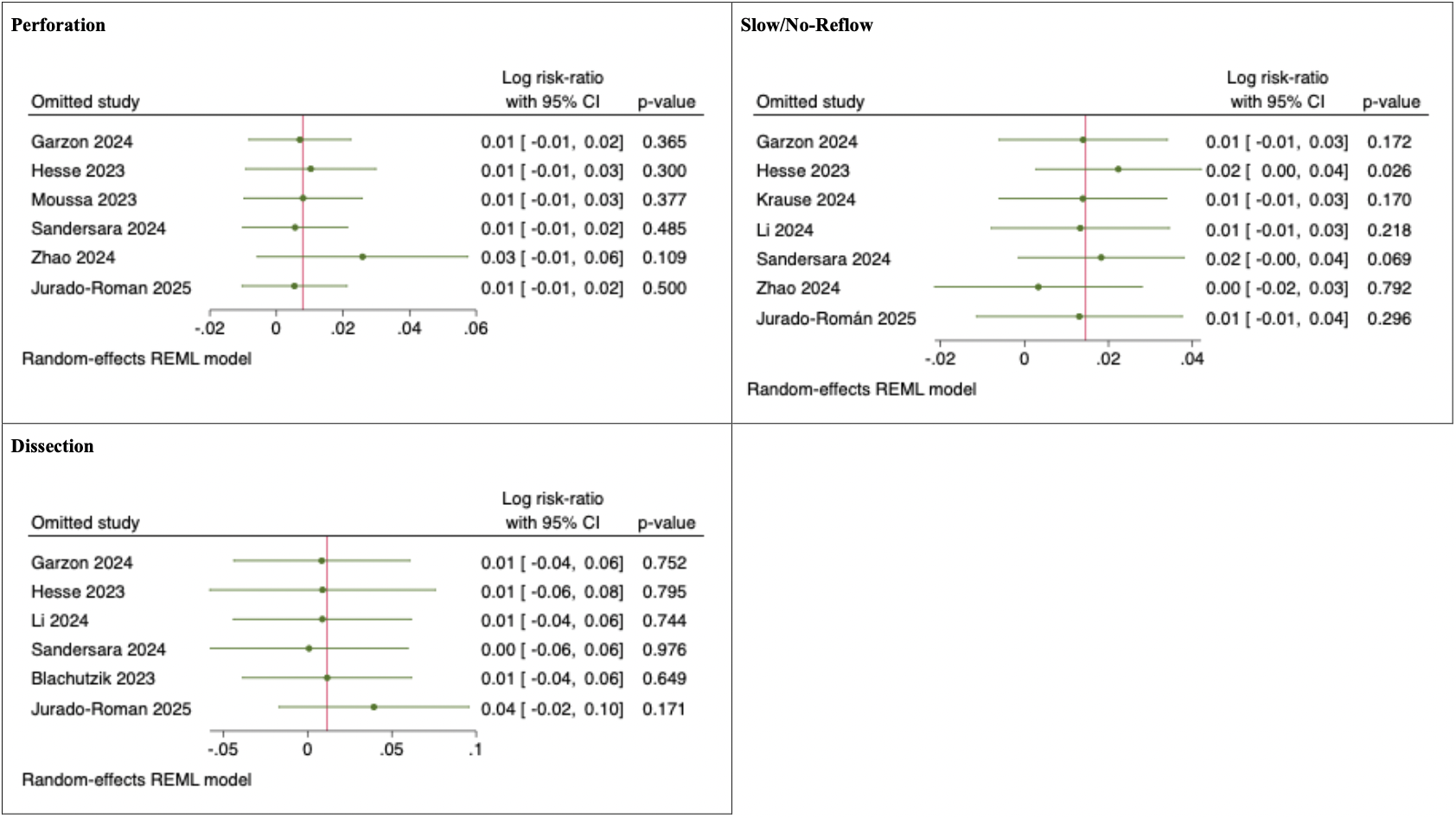
**

**Supplementary Figure 5. Sensitivity Analysis of Procedural Outcomes**

**
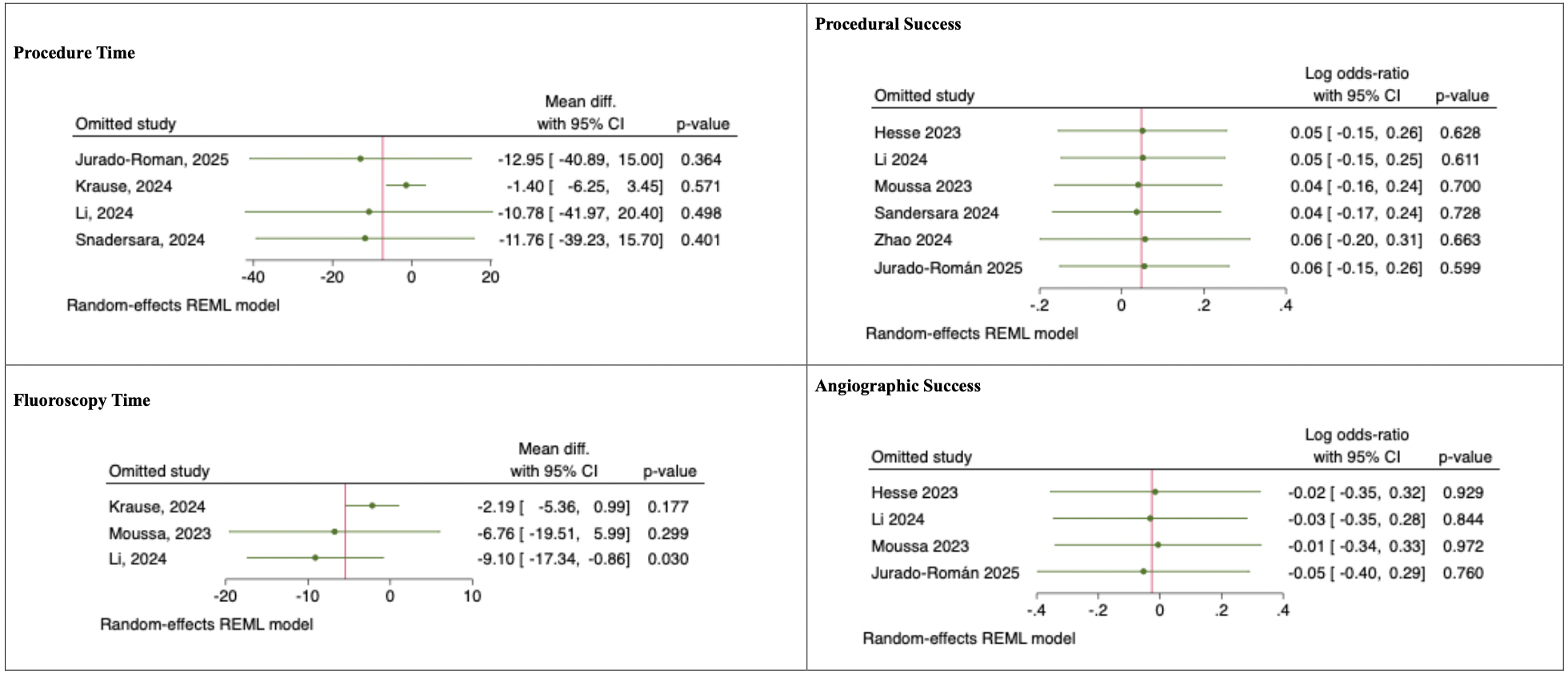
**

**Supplementary Figure 6. Sensitivity Analysis of Dissection based on Study Design**


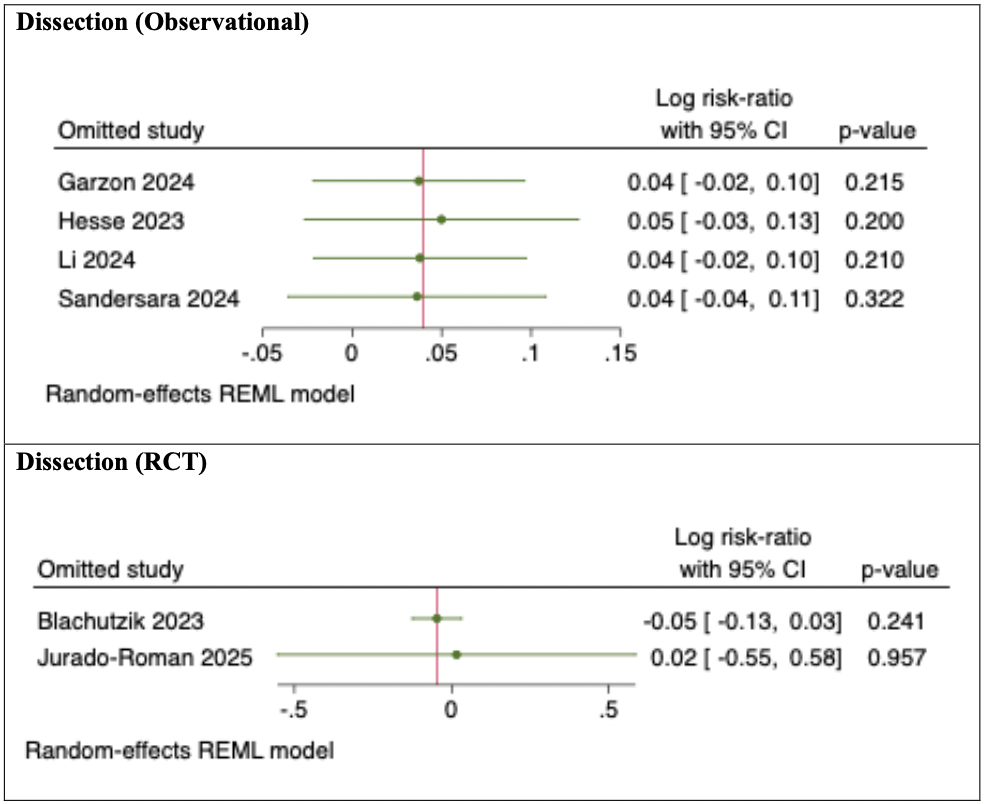


**Supplementary Tables**

**Supplemental Table 1.**

| PICOS | Keywords |
| --- | --- |
| Patients | “Patient with moderate-to-severe calcified atherosclerosis” |
| Intervention | “Intravascular lithotripsy" |
| Comparison | “Rotational atherectomy” |
| Outcomes | “Major adverse cardiac event (MACE)” OR “myocardial infarct” OR “all-cause mortality” OR “dissection” OR “perforation” OR “slow/no reflow” OR “procedural success” OR “fluoroscopy time” OR “procedural time” |
| Study Design | “Observational Study” and “Randomized Controlled Trial” |

**Supplemental Table 2. Newcastle-Ottawa Scale**

Observational-NOS

| **First Author** | **Selection** | | | | Comparability | **Outcome** | | | Total Score | Study Quality |
| --- | --- | --- | --- | --- | --- | --- | --- | --- | --- | --- |
|  | Representativeness of exposed control | Selection of nonexposed control | Ascertainment of exposure | Outcome not present at the start of the study |  | Assessment of outcome | Length of follow-up | Adequacy of follow up |  |  |
| Garzon | * | * | * | * | * | * |  | * | 7 | good |
| Sandersara | * | * | * | * | * | * |  | * | 7 | good |
| Hesse | * | * | * | * | * | * | * | * | 8 | good |
| Moussa | * | * | * | * | * | * | * | * | 8 | good |
| Li | * | * | * | * | * | * | * | * | 8 | good |
| Zhao | * | * | * | * |  | * | * | * | 7 | good |
| Krause | * | * | * | * | * | * | * | * | 8 | good |

**Supplemental Table 3. RoB 2 Tool**

RCT-RoB-2

| First author | Bias from randomization process | Bias due to deviations from intended interventions | Bias due to missing outcome data | Bias in measurement of the outcomes | Bias in selection of the reported result | Overall risk of bias |
| --- | --- | --- | --- | --- | --- | --- |
| Jurado-Román | Low | Some concern | Low | Low | Low | Low |
| Blachutzik | Low | Low | Low | Low | Low | Low |

**Supplementary Table 4. Inclusion and exclusion criteria and definition of outcomes**

| **First Author** | **Inclusion and Exclusion Criteria** | **MACE** | **Procedural and Angiographic Success** | **Moderate-to-Severe Calcification** |
| --- | --- | --- | --- | --- |
|  |  |  |  |  |
| Garzon | Inclusion:  Patient who had undergone coronary stenting using either RA or IVL to treat lesion with severe calcification  Exclusion:  Patient who underwent RA and IVL, patient with poor-quality or absent IVUS images after lesion preparation, patient who had used imaging other than IVUS, patient treated with DCB only. | Cardiac death, myocardial infarction, stroke, hospitalization due to heart failure or target vessel revascularization | Procedural success:  NR  Angiographic success:  NR | Visible radiopacities without cardiac motion before contrast injection on both sides of the vessel in angiography or a calcium arc >180 degree by IVUS. |
| Sandersara | Inclusion:  Patients with severely calcified de novo or post CABG distal LM stenosis who underwent PCI and were treated with IVL or RA  Exclusion:  NR | All‐cause death, nonfatal myocardial  infarction (MI) or need for emergency target vessel revascularization  (TVR). | Procedural success:  Successful stent delivery with less than 30% residual angiographic  stenosis without in‐hospital major adverse cardiac event (MACE).  Angiographic success:  NR | Severe calcification was defined angiographically as radio‐opacities on both sides of the arterial wall before injection of contrast medium or by IVUS as >270° arc of calcium in the distal LM, ostial left anterior descending (LAD) or ostial left circumflex (LCX). |
| Hesse | Inclusion:  Patient with LMCA disease treated by IVL or RA, patient with ACS or electively for treatment for stable angina and were found to have operator-assessed angiographic or intravascular imaging-based evidence of severe coronary artery calcification in a severely stenotic segment.  Exclusion:  Repeat PCI with IVL and/or RA | Cardiac death, myocardial infarction, or target vessel revascularization | Procedural success:  Stent delivery with <50% residual stenosis and without serious clinical complication (in-hospital MACE and peri-procedural acute oedema, cardiac tamponade, arrhythmia, acute kidney injury, major and minor bleeding)  Angiographic success: Stent delivery with <50 % residual stenosis and without serious angiographic complications (dissections, perforations, abrupt vessel closure, persistent slow flow-no reflow and stent/device loss that required further intervention.) | NR |
| Moussa | Inclusion:  Patient with balloon-crossable heavily calcified coronary lesion  Exclusion:  Balloon-uncrossable lesion, combined used of RA and IVL for same target lesion or cardiogenic shock | All-cause mortality, target lesion revascularization, stroke and stent thrombosis | Procedural success:  Angiographic success without in-hospital complications. Major adverse cardiovascular events (MACE) were defined as the composite of all-cause mortality, target lesion revascularization (TLR), stroke and stent thrombosis (ST).    Angiographic success:  In-stent residual stenosis ≤30 % without significant angiographic complications (severe coronary dissection impairing flow [type D-F], perforation, abrupt clo- sure or no-reflow). | Qualitative angiography radiopacities seen without cardiac motion before contrast injection, usually affecting both sides of the arterial lumen radio-opacities noted before contrast injection |
| Li | Inclusion:  Severe calcification of their coronary arteries and the requirement of PCI. Severe calcification encompassed degree III and degree IV calcification  Exclusion:  (1) patients with acute coronary syndrome with ST-segment elevation or cardiogenic shock;  (2) lesions that could not be passed by a guidewire;  (3) lesions that were obviously rich in thrombus;  (4) venous bridging vascular lesions;  (5) coronary vessels with angles >90;  (6) coronary vessels with severe spiral dissection; and  (7) coronary vessels <2.5mm in diameter. | NR | NR | Severe calcification encompassed degree III and degree IV calcification.  In degree III calcification, before cardiac fluoroscopy or contrast injection, coronary vessel shadows and alignment are clearly visible.  In degree IV calcification, before cardiac fluoroscopy or contrast injection, coronary vessel shadows, contours, and alignments are clear. |
| Zhao | Inclusion:  Patient who has severe coronary artery calcification, patient who has received IVL or RA  Exclusion:  Age <18 years old, contraindicated or not suitable for PCI, receiving both IVL and RA | All-cause mortality, non- fatal myocardial infarction (MI), or requiring emergency target vessel revascularization (TVR). | Procedural success:  Successful stent implan- tation, residual vascular stenosis less than 30% and no major adverse cardiac events (MACE) in the hospital.  Angiographic success:  NR | Radiographic opacities on both sides of the arterial wall displayed by fluoroscopy before injection of contrast agent. |
| Jurado-Román | Inclusion:  Men and women >=18 years of age with clinical indications for PCI (chronic or acute coronary syndromes) in vessels with reference diameters >=2.5 and <4.0 mm and moderate to severe calcification estimated by coronary angiography.  Exclusion:  The culprit lesions of ST-segment elevation acute coronary syndrome. cardiogenic shock, inability to tolerate dual antiplatelet therapy for >=6 months for those not on oral anticoagulation, and impossibility to obtain informed consent from the patient or to conduct, atleast, 1 year follow-up | NR | Procedural success:  Angiographic success without serious procedural complications.  Angiographic success:  Final TIMI flow grade 3 and final stenosis <20% | Moderate calcifications were defined as radiopaque densities not only with cardiac motion before a contrast injection (one side of the vessel or both)  Severe calcifications were defined as radiopaque densities observed without cardiac motion. |
| Krause | Inclusion:  Severe coronary artery disease due to complex coronary anatomy, calcified stenoses and corresponding previous illnesses who are not suitable for surgical care and all patients who had an impaired LVEF or one is to be expected to be at risk of hemodynamic compromise during the intervention, based on the characteristics of the lesion.  Exclusion:  Patients age below 18 years, contraindication for pMCS, patients suitable for cardiac surgical treatment, patients receiving Rota-Shock (RA + IVL), and patients in cardiac arrest. Analysis of the lesion characteristics (e.g., length of calcified portion, total length, eccentric, or concentric) was made afterward through a review of the coronary angiography by an operator blinded to the procedure groups. | Cardiac death, stroke, peri-interventional myocardial infarction according to the fourth universal definition of MI | Procedural success:  NR  Angiographic Success:  NR | NR |
| Blachutzik | Inclusion:  Clinically relevant coronary stenosis with proven myocardial ischemia and severe calcification as defined by coronary angiography showing radiopacities noted without cardiac motion before contrast injection, compromising both sides of the arterial lumen.  Exclusion:  True bifurcation lesions requiring 2-stent strategies and patients with cardiogenic shock requiring intravenous catecholamines. | NR | Procedural success:  NR  Angiographic success:  NR | Severe calcification as defined by coronary angiography showing radiopacities noted without cardiac motion before contrast injection, compromising both sides of the arterial lumen. |

**Supplementary Table 5. Characteristics of Included Studies**

| **First Author** | **Year** | **Follow Up** | **Center** | **Study Design** | **Lesion Target** | **Device Type** | | **Imaging Technique** | **Sample Size** | | |
| --- | --- | --- | --- | --- | --- | --- | --- | --- | --- | --- | --- |
|  |  |  |  |  |  | **IVL** | **RA** |  | **IVL** | **RA** | **Total** |
| Garzon[[8]](https://paperpile.com/c/GAk3Vw/O9Z27) | 2024 | 30 days | Single Center (Brazil) | Retrospective Observational | Any Severe Calcification | Shockwave C2 balloon (Shockwave Medical) | RA (Boston Scientific) | IVUS | 12 | 13 | 25 |
| Sandesara[[6]](https://paperpile.com/c/GAk3Vw/s3ZaE) | 2024 | 30 days | Single Center (United States of America) | Retrospective Observational | Severely calcified de novo or post CABG distal LM stenosis | IVL (Shockwave Medical) | RA (Boston Scientific) | IVUS | 50 | 57 | 107 |
| Hesse[[10]](https://paperpile.com/c/GAk3Vw/WVRZ3) | 2023 | 1 year | Single Center (United Kingdom) | Retrospective Observational | Unprotected LMCA and severe coronary artery calcification | Shock C2 balloon-based coronary catheter system (Shockwave Medical) | does not specify | Intravascular Imaging either IVUS or OCT | 44 | 81 | 125 |
| Mousa[[24]](https://paperpile.com/c/GAk3Vw/phpHT) | 2023 | 6 months | Single Center (Netherland) | Retrospective Observational | Severe coronary artery calcification (CAC) | Shockwave Intravascular Lithotripsy Coronary (Shockwave Medical). | Rotablator system (Boston Scientific) | does not specify | 50 | 51 | 101 |
| Li[[23]](https://paperpile.com/c/GAk3Vw/D5Js0) | 2024 | 6 months | Single Center (China) | Retrospective Observational | Severe coronary artery calcification | Shockwave C2 disposable intravascular catheter (Shockwave Medical) | Rotablator TM (Boston Scientific) | IVUS | 30 | 30 | 60 |
| Zhao[[5]](https://paperpile.com/c/GAk3Vw/ANpqq) | 2024 | In hospital only | Single Center (China) | Retrospective Observational | Severe Coronary artery calcification | The Shockwave Medical | Rotational atherectomy (Boston Scientific) | Either IVUS or OCT | 152 | 238 | 390 |
| Jurado-Román[[9]](https://paperpile.com/c/GAk3Vw/S3gJ6) | 2025 | 12 months | Multicenter | RCT | Moderate to severe artery calcification | Shockwave balloon (Shockwave Medical) | RotaPro System (Boston Scientific) | OCT | 57 | 57 | 114 |
| Krause[[25]](https://paperpile.com/c/GAk3Vw/q15fj) | 2024 | N/A | Single Center (Germany) | Retrospective Observational | Severe coronary artery disease due to complex coronary anatomy, calcified stenoses and corresponding previous illnesses who are not suitable for surgical care. | IVL (Shockwave Medical) | RA (Boston Scientific) | does not specify | 11 | 14 | 25 |
| Blachutzik[[26]](https://paperpile.com/c/GAk3Vw/n7kdh) | 2023 | In hospital only | Multicenter | RCT | Clinically significant and severely calcified coronary lesions | IVL (Shockwave Medical) | Does not specify | OCT | 12 | 9 | 21 |

| **Certainty assessment** | | | | | | | **№ of patients** | | **Effect** | | **Certainty** | **Importance** |
| --- | --- | --- | --- | --- | --- | --- | --- | --- | --- | --- | --- | --- |
| **№ of studies** | **Study design** | **Risk of bias** | **Inconsistency** | **Indirectness** | **Imprecision** | **Other considerations** | **Intravascular Lithotripsy** | **Rotational Atherectomy** | **Relative  (95% CI)** | **Absolute  (95% CI)** |  |  |
| **All-cause Mortality - In Hospital Outcome** | | | | | | | | | | | | |
| 5 | non-randomised studies | not serious | not serious | not serious | not serious | strong association |  | | | | ⨁⨁⨁◯  Moderate |  |
| **All-cause Mortality - 6 months outcome** | | | | | | | | | | | | |
| 1 | non-randomised studies | not serious | not serious^a^ | not serious | not serious | strong association |  | | | | ⨁⨁⨁◯  Moderate^a^ |  |
| **All-cause Mortality - 1 year outcome** | | | | | | | | | | | | |
| 1 | non-randomised studies | not serious | not serious^a^ | not serious | not serious | none |  | | | | ⨁⨁◯◯  Low^a^ |  |
| **All-cause Mortality - Total** | | | | | | | | | | | | |
| 5 | non-randomised studies | not serious | not serious | not serious | not serious | strong association |  | | | | ⨁⨁⨁◯  Moderate |  |
|  |  |  |  |  |  |  |  |  |  |  |  |  |
